# Massive pulmonary embolism with ST-segment elevation mimicking an isolated right ventricular myocardial infarction in a patient with COVID-19 pneumonia: Case report

**DOI:** 10.1016/j.amsu.2022.104943

**Published:** 2022-11-17

**Authors:** Mohammed El-Azrak, Mohammed Boutaybi, Fadoua Mouedder, Noha EL Ouafi, Nabila Ismaili

**Affiliations:** a Department of Cardiology, Mohammed VI University Hospital, Faculty of Medicine and Pharmacy of Oujda, Mohammed First University, Oujda, Morocco; b Epidemiological Laboratory of Clinical Research and Public Health, Faculty of Medicine and Pharmacy of Oujda, Mohammed First University, Oujda, Morocco

**Keywords:** Pulmonary embolism, Right ventricular infarction, ST-Segment elevation, COVID 19 pneumonia

## Abstract

Pulmonary embolism (PE) is a great simulator; It mimics step by step its main differential diagnosis which is myocardial infarction. Its clinical and electrical manifestations are unspecific. Rarely, an ST-segment elevation can occur making the diagnosis more difficult. Recognizing such an uncommon electrocardiographic (ECG) pattern is of an important relevance to lead to a prompt and suitable therapeutic management. In our paper, we discuss a 68 years-old man case who presents with dyspnea and chest pain with ST-segment elevation in V1, aVR, DIII, and right-sided leads suggestive of isolated right ventricular infarction, admitted in a stable hemodynamical status which rapidly deteriorated. Echocardiographic assessment has shown signs of acute pulmonary heart disease with the presence of the specific McConnell's sign. A computed tomography pulmonary angiogram was performed revealing massive bilateral PE that benefited from thrombolytic therapy with alteplase with a remarkable following and regression of the ST-segment elevation. To our knowledge, this is the first case report of massive PE presenting with these ECG findings in the context of COVID 19 pneumonia, of which practitioners should be aware to better orient diagnosis and therapeutic management.

## Highlights

• Pulmonary embolism can rarely present with an ST-segment elevation.
• We discuss a case of patient who presents with electrographic aspect suggestive of isolated right ventricular infarction that was shown to be a massive pulmonary embolism (PE).
• This is the first case report of massive PE presenting with these ECG findings in the context of COVID 19 pneumonia.

## 1 Introduction and importance

Pulmonary embolism (PE) mimicking an ST-segment elevation in leads looking at the right ventricle (RV) is a very rare entity. The awareness of this electrocardiographic (ECG) pattern in PE should incite an earlier performance of bedside echocardiography that is crucial for diagnostic orientation, which should then lead to computed tomography (CT) pulmonary angiogram (CTPA) performance to confirm PE in order to avoid a useless coronary angiography. Early diagnosis reduces the mortality associated with massive PE whose rate is of 25–65% despite thrombolytic therapy [1]. In our article, we present a case of 68 years-old man with undiagnosed COVID-19 pneumonia infection, who presented with an acute coronary syndrome picture with ST-segment elevation in V1, aVR, DIII, and right-sided leads suggestive of right ventricular myocardial infarction (RVMI), that proved to be massive bilateral PE. Practitioners must be aware of this uncommon ECG pattern of PE to avoid serious outcomes secondary to delayed diagnosis.

This case report has been reported in line with the SCARE 2020 criteria [2].

## 2 Case presentation

**Fig. 1:**
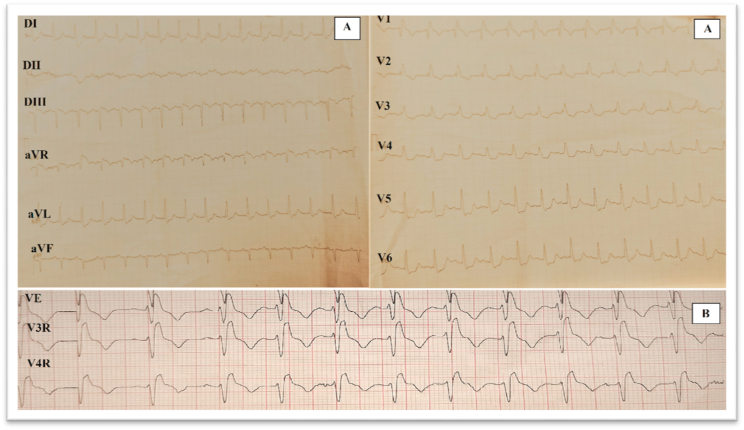
**A** ECG showing ST-segment elevation in V1, aVR and DIII leads, ST-segment depression in septo-apico-lateral leads and T-waves inversions in V1–V3 leads. **B:** Right-sided ECG showing ST-segment elevation in V3R and V4R leads suggestive of right ventricular myocardial infarction.

A 68 years old man presented to our emergency room for acute dyspnea and chest pain evolving for 3 h. His past medical history is significant for diabetes mellitus treated with metformin 850 mg per day, active tobacco use, overweight and abdominal obesity, with no past history of recent surgery or immobilization, cancer, or previous deep venous thrombosis. The patient complains of dyspnea and chest tightness for 3 h without cough, nor hemoptysis or expectoration. On presentation, clinical exam found a conscious patient, hemodynamically stable with tachycardia at 120 beats per minute, tachypnea at 24 cycles per minute. The oxygen saturation was at 84% in room air and 94% under 8 L per minute of Oxygen. Cardiac and pulmonary examinations were normal. A 18-leads resting electrocardiogram shows sinus tachycardia with heart rate of 124 beats per minute, left axis deviation, right bundle branch block (RBBB) with ST-segment elevation in V1, aVR, DIII, V3R and V4R leads, ST-segment depression in septo-apico-lateral leads and T-waves inversions in V1–V3 leads [Fig. 1,A]. A diagnosis of isolated RVMI was then suggested [Fig. 1,B], but the RBBB in presence of dyspnea, oxygen desaturation, sinus tachycardia and normal pulmonary exam was also suspicious of PE.

**Fig. 2:**
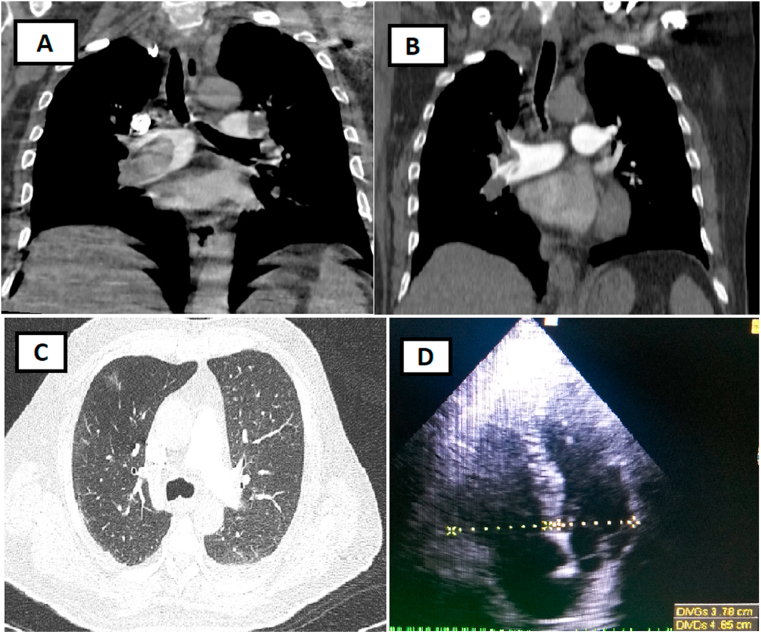
CT pulmonary angiography showing **A:** Pulmonary artery thrombosis extended to its right and left branches with total obstruction of their lumens **B:** Residual right lobar and segmental PE with left segmental PE 48 hours after thrombolytic therapy. **C:** Bilateral peripheral ground-glass opacities classified CORADS 4. **D:** Apical 4 chamber view of echocardiogram showing dilated right ventricle (RV) and septal deviation due to pressure overload in RV.

Therefore, a bedside transthoracic echocardiography was performed, showing normal global and segmental left ventricular systolic function, with paradoxical interventricular septal motion, dilated RV [Fig. 2,D] with systolic dysfunction (tricuspid annular plane systolic excursion (TAPSE): 14mm; S wave: 0,09 m/s) and global hypokinesis sparing the apex which is hypercontractile (McConnell's sign), and RV systolic pressure to be around 30 mm Hg, suggesting the diagnosis of PE. We performed a CTPA showing a CT image of bilateral pulmonary embolism at the level of pulmonary artery extended to its right and left branches with total obstruction of their lumens [Fig. 2,A]. It shows also bilateral, peripheral and lower ground-glass opacities suggesting mild viral pneumonia due to COVID-19 classified CORADS 4 [Fig. 2,C]. While he was in the emergency room, an acute deterioration of hemodynamic status occurred with a drop of systolic blood pressure to 80 mmHg. Massive bilateral PE secondary to COVID-19 pneumonia infection was retained and thrombolytic therapy with intravenous alteplase was administered (10mg in 30 min) followed by infusion of 90mg in 2 hours with a prominent clinical improvement; The blood pressure was again within normal ranges, the dyspnea disappeared, and the oxygen saturation improved to 94% under 2 L per minute of Oxygen. At laboratory assessment, troponin serum level was elevated (peak 311 ng/mL; normal value: <26 ng/ml). Other results are summarized in [Table 1]. The patient was discharged off Oxygen therapy 2 days later and was started on Acenocoumarol 2mg/day with a target international normalized ratio (INR) of 2–3 for six months. A Doppler ultrasound of lower limbs did not reveal deep venous thrombosis and PCR test was negative with negative anti-SARS-CoV-2 IgM and positive IgG antibodies. Tumor biomarkers were negative. Serial echocardiographic exams have shown a RV systolic function improvement with a remained dilated RV and control CT has shown residual right lobar and segmental PE with left segmental PE [Fig. 2,B]. The patient was discharged after six days with oral anticoagulation and COVID-19 treatment.

**Table 1:** Significant laboratory findings.

Examen	Results	Normal Values
Albumin	43.00	34–54
C-reactive protein (mg/l)	7.27	6–12
Urea (g/l)	0.24	<0.45
Creatinine (mg/l)	13.52	(6-12)
Potassium (mmol/l)	3.7	(3–5)
Natremia (mmol/l)	138	(135–140)
Troponin Level (ng/ml)	**311,7**	**< 26**
Prothrombin	73	(70–100)
White blood cells (E/mm3)	17,080	(4000–10,000)
Hemoglobin (g/dl)	17.1	>13
Hematocrit	51.2	40–52
Platlets	178,000	(150,000–400,000)

## 3 Clinical discussion

PE and RVMI are known to be two of the most challenging differential diagnoses. PE is a common disease with a mortality rate of 7%–11% [3], while isolated RVMI is very rare and found in less of than 3% of all myocardial infarction cases [4]. Among etiologies of both, COVID-19 is now widely incriminated. This one affects coagulation and causes both arterial and venous thrombo-embolism [5]. A large meta-analysis assembling 20 studies found a weighted mean prevalence of 18.9% for PE in patients with COVID-19 [6].

Symptoms for PE and RVMI can be similar and are unspecific for one or another disease. Typically, most frequent PE clinical manifestations include dyspnea (79%), palpitations (26%), chest pain (47%) and signs of pulmonary hypertension and right ventricular failure (47%) [7]. They can only be useful when associated with the clinical context. Electrocardiogram offer additional signs to differentiate the two diagnoses in 30–40% of cases [8]. Typical findings of PE are sinus tachycardia, S1Q3T3 pattern, rightward axis shift, incomplete or complete RBBB, and T-wave inversions in V1–V3/V4 leads [9]. On the other hand, RVMI occurs often in the presence of inferior wall changes [10], and is often misinterpreted as an anterior left ventricular wall infarction because of ST changes in V1–V3 leads. Isolated RVMI shows ST elevation in V1 and aVR leads which is more marked in aVR [11,12], and right-sided V3R and V4R leads may guide the diagnosis showing ST elevation or Q waves, since V4R ST elevation is of 100% sensitivity, 87% specificity, and 92% predictive accuracy for RVMI [13]. ST segment elevations are unusual in PE, and when present, they concern V1–V3 or V4 leads as a marker of RV strain, as reported in many series [14]. In our case, PE ECG signs are so atypical and was literally mimicking a RVMI ECG pattern with ST-segment elevations in V1, aVR, DIII, V3R and V4R leads. For our best knowledge, this is the first case reporting this ECG pattern for PE. We suggest that ST-segment elevation is due to acute RV ischemia related to the elevation in right ventricular afterload causing its dilation. Ischemia could also result from raised catecholamines rate, hypoxia or hypotension [15].

Serum troponin elevation may be observed with either diagnoses due to RV ischemia. Douketis and al. Reported a raised troponin level in 20.8% of massive PE cases with low sensitivity and specificity [16]. On transthoracic echocardiography, findings are also unspecific and may include dilated right cardiac chambers, RV wall motion abnormalities, paradoxical movement of interventricular septum, right ventricular systolic dysfunction, increased systolic pulmonary artery pressure and dilated inferior vena cava [17]. One important distinguishing sign is McConnell's sign consisting of RV free wall severe dyskinesia sparing the RV apex with 77% sensitivity and 94% specificity [18]. In our case, the presence of this sign was alarming for the PE diagnosis and incited us to perform a pulmonary CT angiography. Moreover, CT angiography is still the tool of choice for the visualization of intraluminal thrombi pulmonary arteries with high sensitivity [19].

Once the diagnosis of PE is made, rapid risk stratification is mainly based on hemodynamic status, pulmonary embolism severity index (PESI), cardiac biomarkers (troponins I and T, Brain natriuretic peptides) and echocardiographic findings of RV strain [20]. Our patient was hemodynamically unstable an hour after presentation, with massive PE. Urgent fibrinolysis was administered with a good evolution noticed 1 h later.

## 4 Conclusion

This case report highlights the importance of recognizing PE as differential diagnosis of isolated RVMI presenting with ST-segment elevation in leads looking at the RV. The right diagnosis should be aided by bedside echocardiography performance and CT pulmonary angiography, which averts the patient a useless coronary angiography and guide a prompt therapeutic strategy.

## Ethical approval

The patient approval has been given.

The ethics committee agreement has been given too.

## Sources of funding

No sources of funding

## Author contribution

Project administration: Nabila ISMAILI.

Conceptualization and supervision: Noha EL OUAFI.

Data collection: Mohammed BOUTAYBI.

Data analysis, Writing – original draft: Mohammed EL-AZRAK.

Review and editing: Fadoua MOUEDDER

## Registration of research studies

Name of the registry:

Unique Identifying number or registration ID:

Hyperlink to your specific registration (must be publicly accessible and will be checked):

## Guarantor

MOHAMMED EL-AZRAK.

## Consent

Written informed consent was obtained from the patient for publication of this case report and accompanying images. A copy of the written consent is available for review by the Editor-in-Chief of this journal on request.

## Provenance and peer review

Not commissioned, externally peer-reviewed.

## Declaration of competing interest

Authors declare no conflicts of interest.

## References

[1] Turetz M., Sideris A.T., Friedman O.A., Triphathi N., Horowitz J.M. (2018 Jun). Epidemiology, pathophysiology, and natural history of pulmonary embolism. Semin. Intervent. Radiol..

[2] Agha R.A., Franchi T., Sohrabi C., Mathew G. (2020). For the SCARE group. The SCARE 2020 guideline: updating consensus surgical CAse REport (SCARE) guidelines. Int. J. Surg..

[3] Laporte S., Mismetti P., Décousus H., Uresandi F., Otero R., Lobo J.L., Monreal M., Investigators R.I.E.T.E. (2008 Apr 1). Clinical predictors for fatal pulmonary embolism in 15,520 patients with venous thromboembolism: findings from the Registro Informatizado de la Enfermedad TromboEmbolica venosa (RIETE) Registry. Circulation.

[4] Wartman W.B., Hellerstein H.K. (1948 Jan). The incidence of heart disease in 2,000 consecutive autopsies. Ann. Intern. Med..

[5] Bikdeli B., Madhavan M.V., Jimenez D., Chuich T., Dreyfus I., Driggin E., Nigoghossian C., Ageno W., Madjid M., Guo Y., Tang L.V., Hu Y., Giri J., Cushman M., Quéré I., Dimakakos E.P., Gibson C.M., Lippi G., Favaloro E.J., Fareed J., Caprini J.A., Tafur A.J., Burton J.R., Francese D.P., Wang E.Y., Falanga A., McLintock C., Hunt B.J., Spyropoulos A.C., Barnes G.D., Eikelboom J.W., Weinberg I., Schulman S., Carrier M., Piazza G., Beckman J.A., Steg P.G., Stone G.W., Rosenkranz S., Goldhaber S.Z., Parikh S.A., Monreal M., Krumholz H.M., Konstantinides S.V., Weitz J.I., Lip G.Y.H. (2020). Global COVID-19 thrombosis collaborative group, endorsed by the ISTH, NATF, ESVM, and the IUA, supported by the ESC working group on pulmonary circulation and right ventricular function. COVID-19 and thrombotic or thromboembolic disease: implications for prevention, antithrombotic therapy, and follow-up. JACC state-of-the art review. J. Am. Coll. Cardiol..

[6] Di Minno A., Ambrosino P., Calcaterra I., Di Minno M.N.D. (2020 Oct). COVID-19 and venous thromboembolism: a meta-analysis of literature studies. Semin. Thromb. Hemost..

[7] Stein P.D., Beemath A., Matta F. (2007). Clinical characteristics of patients with acute pulmonary embolism: data from PIOPED II. Am. J. Med..

[8] Ginghina C., Caloianu G.A., Serban M., Dragomir D. (2010 Jul-Sep). Right ventricular myocardial infarction and pulmonary embolism differential diagnosis--a challenge for the clinician. J Med Life.

[9] Falterman T.J., Martinez J.A., Daberkow D., Weiss L.D. (2001 Oct). Pulmonary embolism with ST segment elevation in leads V1 to V4: case report and review of the literature regarding electrocardiographic changes in acute pulmonary embolism. J. Emerg. Med..

[10] Geft I.L., Shah P.K., Rodriguez L. (1984). ST elevation in Downloaded from ang.sagepub.com at BRIGHAM YOUNG UNIV on 16, 2015 124 leads V1 to V5 may be caused by right coronary artery occlusion and acute right ventricular infarction. Am. J. Cardiol..

[11] Turkoglu S., Erden M., Ozdemir M. (2008). Isolated right ventricular infarction due to occlusion of the right ventricular branch in the absence of percutaneous coronary intervention. Can. J. Cardiol..

[12] Finn A.V., Antman E.M. (2003). Images in clinical medicine. Isolated right ventricular infarction. N. Engl. J. Med..

[13] Somers M.P., Brady W.J., Bateman D.C., Mattu A., Perron A.D. (2003 Nov). Additional electrocardiographic leads in the ED chest pain patient: right ventricular and posterior leads. Am. J. Emerg. Med..

[14] Omar H.R. (2016 Dec). ST-segment elevation in V1-V4 in acute pulmonary embolism: a case presentation and review of literature. Eur. Heart J Acute Cardiovasc. Care.

[15] Falterman T.J., Martinez J.A., Daberkow D., Weiss L.D. (2001 Oct). Pulmonary embolism with ST segment elevation in leads V1 to V4: case report and review of the literature regarding electrocardiographic changes in acute pulmonary embolism. J. Emerg. Med..

[16] Douketis J.D., Crowther M.A., Stanton E.B. (2002). Elevated cardiac troponin levels in patients with submassive pulmonary embolism. Arch. Intern. Med..

[17] Lindqvist P., Calcutteea A., Henein M. (2008). Echocardiography in the assessment of right heart function. Eur. J. Echocardiogr..

[18] Casazza F., Bongarzoni A., Capozi A., Agostoni O. (2005 Jan). Regional right ventricular dysfunction in acute pulmonary embolism and right ventricular infarction. Eur. J. Echocardiogr..

[19] Torbicki A., Perrier A. (2008). Guidelines on the diagnosis and management of acute pulmonary embolism. Eur. Heart J..

[20] Konstantinides S.V., Meyer G., Becattini C. (2020). ESC Scientific Document Group: 2019 ESC Guidelines for the diagnosis and management of acute pulmonary embolism developed in collaboration with the European Respiratory Society (ERS): the Task Force for the diagnosis and management of acute pulmonary embolism of the European Society of Cardiology (ESC). Eur. Heart J..

